# COVID-19: clinical issues from the Japan Surgical Society

**DOI:** 10.1007/s00595-020-02047-x

**Published:** 2020-07-11

**Authors:** Masaki Mori, Norihiko Ikeda, Akinobu Taketomi, Yo Asahi, Yoshio Takesue, Tatsuya Orimo, Minoru Ono, Takashi Kuwayama, Seigo Nakamura, Yohei Yamada, Tatsuo Kuroda, Kenji Yuzawa, Taizo Hibi, Hiroaki Nagano, Michiaki Unno, Yuko Kitagawa

**Affiliations:** 1 The Japanese Surgical Society, Tokyo, Japan; 2 Committee for novel coronavirus disease 2019 outbreak of the Japanese Surgical Society, Tokyo, Japan; 3 Department of Surgery and Science, Graduate School of Medical Sciences, Kyushu University, 3-1-1 Maidashi, Higashi-ku, Fukuoka, 812-8582 Japan; 4 Department of Surgery, Tokyo Medical University, 6-7-1 Nishishinjuku, Shinjuku-ku, Tokyo, 160-0023 Japan; 5 Department of Gastroenterological Surgery I, Hokkaido University Graduate School of Medicine, Kita-ku, Kita 15, Nishi 7, Sapporo, Hokkaido 060-8638 Japan; 6 Department of Infection Control and Prevention, Hyogo College of Medicine, 1-1, Mukogawa-cho, Nishinomiya, Hyogo 663-8501 Japan; 7 Department of Cardiovascular Surgery, Graduate School of Medicine, The University of Tokyo, 7-3-1 Hongo, Bunkyo-ku, Tokyo, 113-8655 Japan; 8 Department of Breast Surgical Oncology, Showa University School of Medicine, 1-5-8, Hatanodai, Shibagawa-ku, Tokyo, 142-8666 Japan; 9 Department of Pediatric Surgery, Keio University School of Medicine, 35 Shinanomachi, Shinjuku-ku, Tokyo, 160-8582 Japan; 10 Department of Transplantation Surgery, National Hospital Organization Mito Medical Center, Ibaraki-machi, Higashiibaraki-gun, Ibaraki, 311-3193 Japan; 11 Department of Pediatric Surgery and Transplantation, Kumamoto University Graduate School of Medical Sciences, 1-1-1 Honjo, Chuo-ku, Kumamoto, Kumamoto 860-8556 Japan; 12 Department of Gastroenterological, Breast and Endocrine Surgery, Yamaguchi University Graduate School of Medicine, 1-1-1 Minami-Kogushi, Ube, Yamaguchi 755-8505 Japan; 13 Department of Surgery, Tohoku University Graduate School of Medicine, 1-1, Seiryo-machi, Aoba-ku, Sendai, 980-8574 Japan; 14 Department of Surgery, Keio University School of Medicine, 35 Shinanomachi, Shinjuku-ku, Tokyo, 160-8582 Japan

**Keywords:** Novel coronavirus disease 2019 (COVID-19), Surgical triage, Aerosol-generating procedures (AGPs), Personal protective equipment (PPE)

## Abstract

In this unprecedented COVID-19 pandemic, several key issues must be addressed to ensure safe treatment and prevent rapid spread of the virus and a consequential medical crisis. Careful evaluation of a patient’s condition is crucial for deciding the triage plan, based on the status of the disease and comorbidities. As functionality of the medical care system is greatly affected by the environmental situation, the treatment may differ according to the medical and infectious disease circumstances of the institution. Importantly, all medical staff must prevent nosocomial COVID-19 by minimizing the effects of aerosol spread and developing diagnostic and surgical procedures. Polymerase chain reaction (PCR) screening for COVID-19 infection, particularly in asymptomatic patients, should be encouraged as these patients are prone to postoperative respiratory failure. In this article, the Japan Surgical Society addresses the general principles of surgical treatment in relation to COVID-19 infection and advocates preventive measures against viral transmission during this unimaginable COVID-19 pandemic.

## Introduction

The rapid spread of the novel coronavirus disease 2019 (COVID-19), which appeared first in Wuhan, China, has caused a worldwide pandemic that has reached Japan [1–3]. Facing the COVID-19 pandemic outbreak, the medical environment for Japanese surgeons has changed dramatically, presenting unprecedented challenges to our routine clinical practice. Our most important task is to prevent the medical care breakdown until the COVID-19 pandemic subsides. Dealing with the COVID-19 pandemic has placed a great burden on health care capacity, and breakdown of medical care will occur if this burden overwhelms the existing health capacity. In the event of breakdown of medical care, the hospital system will stop functioning normally, resulting in more COVID-19-related deaths. In this situation, other patients needing care, such as those with chronic disease, cancer, or trauma, are also confronted by a lack of medical resources. This secondary outcome of the pandemic could result in a further increase in deaths of these patients.

## Elective surgery during the COVID-19 pandemic

In this critical situation, the surgeon faces two issues: Appropriate triage of surgery and prevention of nosocomial infection. To resolve these two issues, “Proposal for surgical-related procedure for novel coronavirus disease 2019 positive or suspected patients” was issued in April 1st, 2020 at the suggestion of the committee for COVID-19 of the Japanese Surgical Society, and with the approval of 12 other surgical societies [4].

Performing triage of surgery during the COVID-19 pandemic is necessary for two reasons. First, there is a high risk of deterioration of COVID-19 during the post- or intraoperative period for patients undergoing surgery during the incubation period of COVID-19. A report from Wuhan, China analyzing the clinical data of 34 patients who underwent elective surgery during the incubation period of COVID-19 revealed that COVID-19 pneumonia developed in all 34 (100%), with accompanying abnormal findings on computed tomography studies, shortly after the surgery. Fifteen of these patients (44.1%) required intensive care unit (ICU) management for worsening COVID-19 symptoms and seven (20.5%) died in ICU [5]. The second reason is the system-wide shortage of medical resources during the COVID-19 pandemic. Mainly by respiratory failure, almost 30% of COVID-19 patients become severely unwell, mainly because of respiratory failure, necessitating invasive mechanical ventilation or assistance with extracorporeal membrane oxygenation (ECMO) [6]. Vast medical resources including specialist staff, ICU beds, and personal protective equipment (PPE) are consumed in the ICU setting, even during normal operations, and this amount is accentuated in the setting of the COVID-19 pandemic, because of the increased risk of nosocomial infection. The ability of hospital system to manage both COVID-19 patients and general clinical patients, including those with organ failure or cancer, simultaneously, must be maintained. Thus, careful utilization of these limited medical resources is required to balance the needs of routine clinical situations and COVID-19 patients, to prevent breakdown of the medical system. Proficient surgical triage is required for the surgeon to utilize the medical resources more efficiently and effectively.

Table 1 outlines the “Guideline for performing surgical triage during the COVID-19 pandemic”. In this guideline, status of the medical care system is classified into a stable period and a restricted period, separating patients with and those without novel coronavirus infection, and disease for surgery into three levels: A, disease that is nonfatal or does not require urgent medical intervention; B, disease that is not immediately fatal but may potentially be fatal or at risk of becoming severe; and C, disease that may be fatal within days–months without surgical intervention. The decision for surgical planning about whether to perform or postpone surgery is made in each category separated by the classifications described above. In performing triage of surgery during the real clinical practice, this proposal should be implemented with some flexibility since all patients have variations of pathophysiology, progression of illness, and environmental situations, and the correct clinical decisions can only be made after assessing these variations. For instance, even for the same stage of the same disease, the clinical decision to perform or postpone surgery differs when variations in comorbidity, age, and other factors are considered. We should never forget that the attending surgeon, who is usually well informed about the clinical information of each patient, is the one who should be responsible for making the correct decision in surgical triage.

**Table 1:** Guidelines for performing surgical triage during the COVID-19 pandemic (The Japan Surgical Society)

Status of the medical care system^a^			Stable period		Restricted period	
Status of COVID-19 on target patient^b^			Negative^d^	Positive or suspect	Negative^d^	Positive or suspect
Disease level^c^	A	Disease that is nonfatal, or does not require urgent medical intervention	Perform cautiously under appropriate infection control	Postpone	Postpone	Postpone
	B	Disease that is nonfatal but may potentially be fatal or is at risk to become severe	Perform surgery cautiously under appropriate infection control	Postpone, but if not possible, perform surgery cautiously under full infection control	Postpone if possible	Postpone
	C	Disease that may be fatal in a few days or a few months without any surgical intervention	Perform surgery cautiously under appropriate infection control	Consider alternative treatment options, but if not possible, perform surgery cautiously under full infection control	Consider alternative treatment options, but if not possible, perform surgery cautiously under appropriate infection control	Consider alternative treatment options, but if not possible, perform surgery cautiously under full infection control

^a^Status of the medical care system should be judged comprehensively according to vacant hospital beds, medical staff, PPE, acceptance of COVID-19 patients, announcement of state of emergency, pandemic state of COVID-19 each in the relevant area and medical facility

^b^To diagnosis the status of COVID-19 in a target patient, a polymerase chain reaction (PCR) test is preferable. However, if the PCR test is unaffordable, diagnosis should be made comprehensively according to symptoms for the last 2 weeks, overseas travel history, moving history, history of high-risk contact of a target patient or their family, and chest CT if necessary

^c^Disease level should be determined several times, if necessary, by a medical team with the attending doctor in charge, according to the seriousness or emergency of the disease, degree of necessity for treatment, and condition of the target patient

^d^Infection control should be implemented under an infection management manual proposed in each hospital, keeping in mind that confirming a diagnosis may be difficult in some patients with subclinical infection and an inevitable false-negative result of the PCR test for COVID-19

The present proposal also outlines the prevention of nosocomial COVID-19 infection, including via aerosol-generating procedures (AGPs) and PPE. Aerosols are generated through medical procedures such as intubation, extubation, tracheotomy, mask ventilation, bronchoscopy, chest drainage, gastrointestinal endoscopy, electrosurgical procedures for digestive organs, and laparoscopic abdominal surgery. All medical personnel, especially surgeons and anesthesiologists, who are involved in surgery, should be aware of the higher risk of COVID-19 infection from contaminated aerosols generated by these AGPs and implement optimal protection against exposure to aerosols [7, 8]. When surgery or other procedures are performed in patients with confirmed or suspected COVID-19, the medical personnel must protect themselves by wearing PPE. While performing high-risk AGPs, higher-level protection is recommended by wearing respirator masks such as an N95 mask, face shields, and gowns [9]. Medical resources including sophisticated PPE are expected to be short in supply during the COVID-19 pandemic; so, medical facilities are advised to use these resources efficiently by securing stock or considering re-use of the resources where practicable and safe.

To address the COVID-19 pandemic, every surgeon must be informed about the pathology of COVID-19 to be optimally protected against the infection and carry out appropriate treatment for every patient, depending on the specifics of their disease. In this article, we address specific proposals for gastrointestinal surgery, hepatobiliary and pancreatic surgery, cardiovascular surgery, respiratory surgery, pediatric surgery, breast and endocrine surgery, and transplantation surgery, outlining the clinical tasks and treatment guidance for each field during the COVID-19 pandemic.

## Gastrointestinal surgery

Severe acute respiratory syndrome coronavirus 2 (SARS-COV-2) enters cells via the angiotensin-converting enzyme 2 (ACE2) receptor, which is widely expressed not only in pulmonary alveolar cells but also in the enterocytes of the intestinal mucosa [10, 11]. Viral RNA can be found in the stools of infected patients [12] and extrapulmonary symptoms arising from the gastrointestinal tract are common [13–15]. Because of the potential risk of virus transmission, special considerations are required for gastrointestinal surgeons.

### Postponement of surgical treatment

In the era of the COVID-19 pandemic, clinicians should consider whether postponement of surgical treatment is possible to prevent nosocomial transmission and maintain health care capacity [16]. Surgery would be delayed for up to 6 months in patients with large or suspicious polyps, hereditary syndromes, dysplasia/carcinoma in situ in biopsy specimens, and incomplete, questionable margins on polypectomy. Deferring surgery should be considered for patients with early cancer in a resected polyp and stage I and II colorectal cancer, if intensive care unit (ICU) beds/ventilator and resources could be needed. However, curative resection should be performed if delaying surgery by more than 3 months will adversely impact tumor and oncologic outcomes. Neoadjuvant chemotherapy or chemoradiation is recommended for high-risk colorectal cancer to delay surgery until the peak of COVID-19 abates and resources return. Emergency surgery is mandatory for patients with cancers causing bleeding requiring transfusion, obstruction, and impending perforation [16, 17]. For patients with colorectal cancer, alternative treatment approaches including neoadjuvant chemotherapy or chemoradiation to delay surgery could be considered. For patients with inflammatory bowel disease, multiple medical options such as steroids, immunomodulators, and biologics, which provide multi-disciplinary non-surgical care, might be the beneficial alternatives to surgery during this pandemic [18].

### Preoperative polymerase chain reaction (PCR) testing

Patients with symptoms suggestive of COVID-19 should be screened by telephone interview 24–48 h prior to admission for surgery, and delaying surgery until 2 weeks after the resolution of symptoms should be considered. However, because of the relatively long incubation period [19] and evidence suggesting that asymptomatic patients can spread COVID-19 [20, 21], it is now important to clarify the COVID-19 status of patients undergoing surgery [17, 22]. Because of the relatively limited availability of SARS-COV-2 PCR testing in Japan, routine screening by PCR would be preferentially conducted at surgery, which increases the risk of generating SARS-COV-2 aerosols. In patients found to be positive for SARS-COV-2 by PCR, elective surgery should be deferred until SARS-COV-2 clearance [17]. Cancer surgery should be reserved for life-threatening situations only. Patients with acute abdomen such as gastrointestinal perforation, ileus causing intestinal ischemia, and complicated appendicitis, do require urgent surgical intervention [23]. For these patients, PCR results would be obtained after surgery; while, chest computed tomography (CT) can be used as a screening tool prior to surgery [17].

### Surgery for patients with COVID-19

COVID-19 complicates the postoperative course with diagnostic challenges and a potentially high mortality. Aminian et al. [24] reported the development of acute respiratory distress syndrome (ARDS) after surgery in two of the four patients, and three patients died during the perioperative period. In contrast, Cai et al. [25] reported that eight patients underwent abdominal surgery, and postoperative recovery was affected only in one critically ill COVID-19 patient. There are three possible situations in which surgeons need to be prepared to encounter COVID-19. First, some patients admitted to hospital for treatment of COVID-19 infection will suffer additional problems that require surgical intervention. Second, patients admitted to a surgical department with any gastrointestinal symptoms, including abdominal pain and diarrhea, may have concomitant COVID-19 infection. Third, postoperative patients may suffer respiratory symptoms, indicating possible hidden or mild disease before surgery or nosocomial transmission. In these cases, appropriate differential diagnosis is important to improve the patient’s outcome and to prevent further transmission. Lymphocytopenia, elevated C-reactive protein (normal for procalcitonin), increased troponin, increased D-dimer, and mild increases of aspartate aminotransferase and alanine aminotransferase levels have been reported in patients with COVID-19 infection [19, 26, 27] and CT examination is essential for a differential diagnosis [28].

If surgical treatment is required for patients with confirmed or suspected COVID-19 infection, several factors should be considered to prevent the infection of medical staff [29]. The hospital should designate a negative-pressure operating room for patients with COVID-19. The scrub team should be equipped with full personal protective equipment (PPE) including N95 respirators, double gloves, face shields, shoe covers, caps, and body protection coveralls/gowns. Staff must take care not to avoid contamination while removing PPE. All personnel other than the anesthesiologist and assistant nurse should leave the room during intubation and extubation, which are high-risk aerosol-generating procedures [30].

### Selection of surgical procedures for patients with COVID 19

Cancer treatment for patients who are COVID-19 positive should be as conservative as possible, using stent placement for stenosing cancer and performing surgery after the resolution of infection. Hartmann’s procedure should be considered for left-sided occlusion or perforation [17, 29]. Although there are concerns regarding SARS-COV-2 release into CO_2_ during laparoscopic or robotic surgery, there is no clear evidence to support open surgery over minimally invasive surgery [16, 17, 29, 31, 32]. However, all efforts should be made to eliminate the risk of staff exposure to aerosolized particles. This can be achieved by minimizing the creation of a plume (reduce power setting) through the use of electrocautery or ultrasonic scalpels, limiting CO_2_ release into the operating room by lowering the pneumoperitoneum pressure, reducing the Trendelenburg position, or deflating the abdomen before retrieving a specimen or before removing the trocars [29, 31, 32].

### Postoperative complications in surgical patients with COVID-19

Surgeons should be prepared to manage a range of complications in patients with COVID-19. Deep vein thrombosis is the major complication after colorectal surgery. Several studies have presented evidence of COVID-19 associated coagulopathy [33–35] and one described a marked increase in D-dimer levels in patients with COVID-19 [36]. The cumulative incidence of thrombotic complications was 31% in critically ill ICU patients with COVID-19, with pulmonary embolism being the most frequent thrombotic complication [37]. In an autopsy study of patients who died of COVID-19, there was a high incidence of deep venous thrombosis (58%) and one-third of the patients had a pulmonary embolism [38]. Thus, anticoagulant prophylaxis should be considered for COVID-19 patients undergoing colorectal surgery [34]. Severe cardiovascular damage has been identified in some COVID-19 patients [39–41] and patients with underlying cardiovascular diseases seem to have an increased risk of succumbing to the disease [36]. Elevated cardiac troponin was reported in COVID-19 patients [39], implicating myocardial injury as a potential pathogenic mechanism contributing to severe illness and mortality. Particular attention should be given to cardiovascular complications in surgical patients with COVID-19.

### Summary

In the era of the COVID-19 pandemic, elective case triage is essential and for patients whose surgical treatment cannot be postponed, several dedicated measures must be implemented to control disease transmission and prevent complications. Because of the relatively long intubation period and potential of asymptomatic carriers, the COVID-19 status of patients should be confirmed before surgery. Appropriate use of PPE is mandatory. Minimizing aerosolization of particles with smoke evacuation should be considered during laparoscopic procedures. After surgery, special attention must be given to preventing thromboembolism and cardiovascular complications developing in patients with COVID-19.

This article reviewed strategies based on the personal experience of medical staff treating patients undergoing gastrointestinal surgery during the peak period of the COVID-19 pandemic. Currently, the peak of COVID-19 appears to have passed and more elective surgery is being resumed. Therefore, it is important to discuss how to move forward while learning from the immediate past to prepare for the next wave of COVID-19.

## Hepato-pancreatico-biliary surgery

The outbreak of coronavirus disease 2019 (COVID-19) caused by the severe acute respiratory syndrome coronavirus 2 (SARS-CoV-2) has had a major impact on the practice of hepato-pancreatico-biliary (HPB) surgery. While HPB surgery is used to treat benign diseases such as acute cholecystitis, it is mainly performed for the removal of malignant tumors and other cancers. However, cancer patients are at a higher risk of severe events than the healthy population if they contract COVID-19 [42]. In general, the prognosis of malignant disease in the HPB surgical field is poor because of the biological aggressiveness of these types of tumor, and the morbidity and mortality rates following HPB surgery are high [43, 44]. The mortality rate is even higher for such patients infected with COVID-19 [45]. Patients with advanced liver disease also represent a vulnerable cohort in relation to COVID-19 as they have an increased risk of infection and/or a severe disease course [46]. The cumulative evidence, thus, indicates that the decision to perform any type of surgery, including HPB surgery, must be made carefully in situations where COVID-19 is widespread.

During the COVID-19 pandemic, the decision to perform HPB surgery should be made in the same way as other surgery. The recommendation of the Society of Surgical Oncology (SSO) in the United States for managing HPB cancers during this pandemic is that surgery should be considered for all patients with aggressive HPB malignancies, including pancreatic adenocarcinoma, gastric cancer, cholangiocarcinoma, duodenal cancer, ampullary cancer, or liver metastasis of colorectal cancer [47]. When systemic chemotherapy is indicated in addition to surgery, neoadjuvant chemotherapy should be considered as a means of delaying surgery if the patient responds to this treatment and can tolerate it. Surgeries for asymptomatic pancreatic NETs, duodenal and ampullary adenomas, GISTs, and high-risk intraductal papillary mucinous neoplasms should also be delayed unless this will affect the resectability of the lesion. Neoadjuvant chemotherapy, ablation, or stereotactic radiosurgery should be used in the place of surgical resection for liver metastases where possible. Ablation or embolization should be considered as an alternative to surgical resection for hepatocellular carcinoma. The American College of Surgeons (ACS) has provided guidance on the management of non-emergency surgical procedures and recommends that surgery for low-risk cancer should be postponed if possible but that it should not be postponed for most other cancers [48]. The ACS has also provided guidelines for the triage and management of patients with pancreatic and periampullary cancer, highlighting the rates of adverse events, prolonged hospitalization, and readmission following surgery as a result of the technical complexity of the required operations, tumor-related factors such as cachexia and malnutrition, and patient-related factors such as age and comorbidities [49]. These adverse events may be associated with especially high rates of perioperative morbidity and mortality when performed in asymptomatic COVID-19-positive patients. Hence, the ACS argues that the need for surgery should be carefully evaluated for each patient during the pandemic in view of the low cure rates of surgery for these malignancies.

An Italian research group has modified the management algorithm for hepatocellular carcinoma (HCC) during the COVID-19 crisis by preferencing locoregional treatments such as ablation therapy over surgical resection to reduce both postoperative stays in the ICU and hospitalization times. This new approach leaves surgery as a salvage option only for cases not achieving a complete radiological response or that are not appropriate for locoregional treatment [50]. The International Liver Cancer Association (ILCA) has also provided guidance on the management of HCC during the pandemic [51]. The ILCA recommendations for the treatment of HCC provide that surgical resections should be limited to patients with a lower risk of decompensation and those without the comorbidities that would increase the risk of severe outcomes from a COVID-19 infection. A French group has proposed new strategies for the practice of digestive and oncological surgery during the pandemic, given the high rate of morbidity and mortality associated with a pancreatectomy [52]. They recommend that interim chemotherapy be considered for pancreatic adenocarcinomas requiring pancreaticoduodenectomy. For pancreatic adenocarcinomas requiring left splenopancreatectomy, they suggest that surgery can be proposed for patients at low operative risk, but must otherwise be deferred with consideration of interim chemotherapy.

In Japan, the target cases for HPB surgery differ slightly from those in the United States or Europe. An example is that hepatectomy is performed commonly for HCC in Japan unlike in Western countries; hence, HPB surgery in Japan during the COVID-19 pandemic should be considered in accordance with the situation on the ground, but should adhere to the aforementioned guidelines. In general, surgery for benign disease and low-risk malignancies should be postponed if possible. For malignant tumors, surgery should be performed according to the oncological needs of each individual patient, with consideration of possible alternative treatments. Surgical procedures with a high risk of complications should be avoided as their management will typically require multiple interventions across different departments, thus expending hospital resources. It should also be recognized that any new policy can be changed depending on the progression of the pandemic and the capacity of the hospital and its staff.

The main complication of COVID-19 is acute respiratory syndrome caused by lung injury, but organs in the HPB regions may also be damaged by this virus. Angiotensin-converting enzyme 2 (ACE2), which acts as a functional receptor for the SARS coronavirus, is present in the biliary and liver epithelial cells and therefore, the liver is a potential target for COVID-19 infection [53]. The incidence of elevated serum liver biochemistry in hospitalized patients with COVID-19 is reported to range from 14 to 53% and liver injury occurs more commonly in those with severe COVID-19 [53, 54]. It remains uncertain whether COVID-19-related liver damage/dysfunction is caused mainly by the viral infection or by coexisting conditions such as the use of potentially hepatotoxic drugs and the presence of a systemic inflammatory response, respiratory distress syndrome-induced hypoxia, and/or multiple organ dysfunction [55]. Whether pancreatic damage is also caused by COVID-19 is not yet clear but has been suggested by some reports [56–58]. The possibility of pancreatic damage by SARS-CoV-2 is further supported by positive ACE2 expression in the pancreas and the high proportion of COVID-19 patients with pancreatic injury [59]. Taken together, HPB surgeons recognize that the organ damage caused by this virus puts infected patients at a higher risk of poor surgical outcomes.

### Triage for HPB surgery

We propose that triage for HPB surgery be done by following the guidelines of the American Hepato-Pancreato-Biliary Association (AHPBA). In this proposal, the status of the medical care system is classified into a stable period and a restricted period (Table 1).

1. Hepatocellular carcinoma

Resection, ablation, and transplantation should be considered in the stable period, as appropriate, and TACE, ablation, or postponing treatment should be considered in the restricted period. For advanced cases including patients with distant metastasis or multiple lobular lesions, TACE, medical therapy, or best supportive care should be considered.

2. Colorectal liver metastasis

Resection or chemotherapy should be considered in the stable period and chemotherapy should be considered in the restricted period.

3. Intrahepatic cholangiocarcinoma

Resection or chemotherapy should be considered in the stable period and chemotherapy should be considered in the restricted period.

4. Perihilar cholangiocarcinoma

Resection should be considered in the stable period and chemotherapy should be considered in the restricted period. Stenting can be considered in both the stable and restricted periods.

5. Distal cholangiocarcinoma

Resection should be considered in the stable period and chemotherapy should be considered in the restricted period.

6. Pancreatic cancer

For resectable pancreatic cancer, resection should be considered in the stable period and neoadjuvant chemotherapy should be considered in the restricted period. For borderline pancreatic cancer, neoadjuvant chemotherapy should be considered in both the stable and restricted periods.

7. Pancreatic IPMN, cysts, and NETs

Postponing treatment should be considered in both stable and restricted periods. Targeted therapy should be considered if NETs present metastatic or progressing characteristics.

## Cardiovascular surgery

The SARS-CoV-2 pandemic has impacted the cardiovascular surgery community worldwide. The rapidly increasing demand to treat COVID-19 patients has forced the healthcare sector to rearrange the prioritization of those who need surgery for cardiovascular surgery. ICU beds are occupied by COIVD-19 patients who require mechanical ventilation or ECMO support in the most affected regions in United States, Europe, and Japan. Some cardiovascular surgeons, fellows, and residents have joined the ICU care team and face the risk of contracting the disease. To share basic knowledge of measures to prevent the transmission of COVID-19 and experiences with its treatment, an international teleconference convened among the Asian Society for Cardiovascular and Thoracic Surgery, the American Association for Thoracic Surgery, European Society for Cardiothoracic Surgery, and the Society of Thoracic Surgeons (STS). Cardiovascular surgery is considered essential because an inadvertent delay may lead to worsening of the disease condition or even death. In this unprecedented pandemic, an individual hospital or surgeon must make a tough decision about deferring or performing scheduled surgery, depending on whether the hospital and human resources are available, and the degree of risk of deferring the surgery.

The American College of Surgeons (ACS) issued “COVID-19: Elective Case Triage Guidelines for Surgical Care” on March 24, 2020, which outlined the general principles for cardiac surgery [60]. In response to the ACS triage guidelines, the COVID-19 Taskforce at STS published a patient triage guideline statement for cardiac surgery in adult [61] and pediatric [62] patients, in early April. The level of triage is stratified into four tiers for adult patients according to inpatient COVID-19 load and the reduction of operative capacity. The same triage recommendations are applied to transcatheter intervention. Alternative percutaneous therapies should be considered if treatment safety and efficacy are thought to be comparable. A follow-up system to monitor symptoms and progression of the disease is mandatory to provide a patient in need with proper treatment in a timely manner.

- *Tier 1* Asymptomatic outpatients and elective interventions are deferred when there is 0–30% inpatient COVID-19 load and mid-range reduction in operative capacity.
- *Tier 2* Asymptomatic outpatients and patients with anatomy and physiology suggesting that delay will have reasonable safety are judged to be deferrable when there is 30–60% inpatient COVID-19 load and moderate reduction in operative capacity.
- *Tier 3* In-patients who cannot be discharged safely without surgical intervention/correction including emergency services are indicated to need essential surgery when there is 60–80% inpatient COVID-19 load and severe reduction in operative capacity.
- *Tier 4* Only emergency services (based on resource availability) are considered to be essential when there is more than 80% inpatient COVID-19 load and minimal operative capacity [61].

The general triage guideline for congenital cardiac surgery is tabulated according to the timing of intervention for various diagnoses, depending on clinical status and other factors [62]. Timing of surgery is divided into emergency, which must be done within 24–48 h of diagnosis when there are adequate resources; urgent, which should be done within 1–2 weeks when there are adequate resources; and high priority elective, which can be done > 2 weeks later when there are adequate resources, with prerequisite that timing for categories is dependent on the resources available, institutional protocols, and other pending cases. A guidance statement on the triage of cardiac surgery patients was published by the Canadian Society of Cardiac Surgeons, who categorized the triage levels into three stages based on the reduction of hospital services available [63]. The elective case triage guidelines for vascular surgery appeared on the ACS homepage on March 24, 2020 [64]. Each disease category is stratified according to the disease conditions (symptomatic/asymptomatic, acute/chronic, critical/stable, size of aneurysm and so on) into four tiers: 1, postpone; 2a, consider postponing; 2b, postpone if possible; and 3, do not postpone.

Cardiovascular surgery programs started to be impacted by the COVID-19 spread from early April in hospitals of densely populated cities in Japan. Elective cases were deferred in some hospitals, and there was a 25–75% reduction in elective surgery in hospitals where a significant number of COVID-19 patients are being treated in ICU and/or where nosocomial infection has been identified. The Japanese Society for Cardiovascular Surgery issued a Statement on SARS-CoV-2 Infection Measures (in Japanese) on the homepage on April 6, 2020, recommending delay of surgery for stable patients when there are insufficient hospital and human resources and transfer of a patient to near-by hospital for surgery that is needed if the resources are not available [65].

A remarkable reduction in blood donations is another concern because some cardiovascular surgery is difficult to perform without transfusion. The Japan Red Cross reported a 30% reduction in blood donations during the period from late March to early April in Japan [66].

## General thoracic surgery

COVID-19 is a highly infectious disease that primarily affects the lungs. The relative risk of smokers suffering severe COVID-19 symptoms is 1.4 (95% CI 0.98–2.00) and their risk of needing intensive care, controlled ventilation, and of death is 2.4 (95% CI 1.43–4.04) compared with non-smokers [67, 68]. The case fatality rate, being the number of deaths of COVID-19-positive patients/the number of COVID-19-positive patients, is significantly higher among patients with advanced age, cardiovascular disease, diabetes, chronic respiratory disease, and hypertension [68, 69]. Lung cancer patients are at a higher risk because of their history of smoking and co-existing diseases. As operative procedures can impair lung function, particular attention should be given to surgery for lung cancer patients.

COVID-19 can be spread in aerosol-generating events during clinical procedures. For thoracic disease patients, double-lumen endotracheal tube placement, and lung and airway surgery are required. Resulting parenchymal lung leaks have great potential to disseminate infection, making lung cancer surgery a high risk for infection. Preoperative examinations such as pulmonary function test, bronchoscopy, and endobronchial ultrasonography, are also aerosol generating.

Three important points must be considered for general thoracic surgery practice during this COVID-19 pandemic.

1. Screening of asymptomatic COVID-19-positive patients

With the global COVID-19 spread, asymptomatic carriers are estimated to comprise 17.9–33.3% of all cases of infection [70, 71]. Nodular or large ground-glass opacity (GGO) and irregular alveolar consolidation on chest CT are typical COVID-19 pneumonitis images [72].

Cases have been reported of patients undergoing lung cancer surgery, who tested PCR-positive for COVID-19 when their symptoms worsened postoperatively [73]. To avoid this, routine preoperative PCR screening is advised to identify asymptomatic patients. Otherwise, serious postoperative complications and COVID-19 transmission to healthcare workers and other patients may ensue.

2. Bronchoscopy

Considering the exposure risk of clinical staff from bronchoscopy, a noninvasive diagnostic procedure can be prioritized. If the pulmonary lesion is strongly suspicious of cancer, surgical resection with intraoperative diagnosis is recommended. However, bronchoscopy is required for suspected invasive cancer because treatment is based on accurate staging and molecular analysis of the biopsy specimen.

3. Triage of operations for thoracic disease

Only those patients who would be compromised by surgical delay should be triaged for surgery; however, it is necessary to minimize disease transmission and preserve human and medical resources. The management approach should be chosen based on tumor histology, stage, biological behavior and the patient’s condition, by multidisciplinary experts, who must decide if surgery should be performed as soon as feasible, if it should be deferred, or if alternative treatment should be considered [74, 75]. Surgeons should also evaluate the possibility of postoperative ICU treatment or mechanical ventilation during COVID-19 prevalence.

During lung surgery, air-leakage prevention is important, and a tissue sealant may be necessary for pleural tears and the staple line to prevent aerosol viral transmission. Rakovich explained detailed procedures for minimizing the risk of aerosol transmission in lung surgery [76].

Outline of the general triage plan by experts:

- Surgery should be performed as soon as feasible for the following conditions:
  Lung cancer with solid or solid-dominant appearance > 2 cm
  - Node-positive lung cancer
  - Post-induction treatment lung cancer
  - Mediastinal tumor invading the surrounding organs
  - Pneumothorax (ineffective drainage)
  - Severe stenosis of the large airway

- Surgery should be deferred for the following conditions:

- Lung cancer with GGO-dominant appearance.
- Lung cancer with solid or solid-dominant appearance < 2 cm.
- Low-grade malignant tumors such as carcinoid.
- Mediastinal tumors without invasion.
- Benign tumors.

- Alternative treatment should be considered for the following conditions:

- Marginally resectable lung cancer.
- Poor PS and/or cardiopulmonary function needing ICU.

## Breast surgery

Healthcare systems, especially hospital resources and staff, are weighted against the COVID-19 pandemic. Although there are differences among regions and each hospital, we cannot provide the usual standard of care to breast cancer patients. Physicians must prioritize treatments and make appropriate medical decisions for breast cancer patients.

The American Society of Breast Surgeons and an international group recommended guidelines for the treatment of breast cancer during the COVID-19 pandemic [77–79]. Priority categories were defined based on the severity of an individual patient’s condition, including comorbidities, and the potential efficacy of treatments. Patients with an immediately life-threatening and unstable condition, or whose prognosis might be altered by treatment delay in the short term, should be categorized as high priority. Patients for whom certain treatment or services can be indefinitely deferred until the pandemic is over without adversely impacting outcomes can be categorized as low priority. The remaining patients are categorized as middle priority, which accounts for the majority of these patients.

1. Surgical procedures

To minimize using operating room resources, we must consider the time required for recovery and reducing the risk of complications. Patients should be triaged for initial alternative therapy such as primary systematic therapy, whenever possible. Neoadjuvant chemotherapy could be considered for triple negative and HER2-positive patients and preoperative endocrine therapy could be considered for ER-positive patients. For patients in the high priority group, surgery would be recommended without delay, as in normal circumstances; for patients in the middle priority group, procedures that would not be considered invasive would be performed if resources are available; and for lower priority patients, operations would be postponed for 3–6 months.

- High priority
  - Drainage of a breast abscess and evacuation of a hematoma.
  - Completion of neoadjuvant chemotherapy.
  - Early isolated loco-regional recurrence.

- Middle priority
  - Surgery upfront is recommended by a multidisciplinary tumor board.
  - Discordant biopsies are likely to be malignant.

- Low priority
  - DCIS.
  - Positive margin after partial mastectomy for invasive cancer.
  - Excision for a benign tumor.
  - Breast reconstruction.
  - Prophylactic mastectomy.

In some hospitals, patients who have surgery planned are routinely tested for COVID-19 infection a few days before admission. If patient is COVID-19 positive, the operation would be delayed for a few weeks.

2. Systemic therapy

When adjuvant/neoadjuvant chemotherapy is recommended, the appropriate drugs should be available as in normal circumstances. During the COVID-19 pandemic, the number of hospital visits is limited because of the immunosuppression caused by the therapy. Granulocyte colony-stimulating factor should be used routinely as prophylaxis after anthracycline and taxanes in the adjuvant/neoadjuvant setting. Adjuvant endocrine therapy should be continued, but to decrease the number of visits, physicians consult via telemedicine as much as possible. For metastatic breast cancer, therapy should be continued, but physicians must proceed carefully.

3. Diagnostic imaging

Diagnostic imaging and core needle biopsy should be done for patients with a lesion that is strongly suspected to be malignant (BI-RADS category 4 or 5) or local recurrence. (high priority) Metastasis should be evaluated before primary therapy if resources allow. (middle priority) Screening mammography and ultrasound, annual check after surgery for breast cancer, and restaging for asymptomatic metastatic breast cancer should be postponed until after COVID-19.

Breast cancer is a relatively slower growing than other cancers, treatment should be multidisciplinary, and the patient’s preference should be considered as much as possible.

## Pediatric surgery

In the middle of March, 2020, about 2 months after COVID-19 was initially identified in Japan, the second wave of COVID-19 hit Japan. In the midst of this crisis, we summarize, from the pediatric surgeon’s standpoint, the current available data on COVID-19 in the pediatric population and share our experience of clinical practice in Tokyo, Japan, as of April 2020.

### How COVID-19 affects the general pediatric population

Evidence has been accumulating on how COVID-19 affects the pediatric population. Reports based on relatively large-scale data from China (2135 cases, including presumed cases) [80], the US (2572 confirmed cases) [81] and Japan, demonstrate that children under 18 years of age accounted for 1–3% of all COVID-19 cases and that their prognosis is generally good. However, some pediatric patients became critically ill. In particular, infants under 1 year of age seem to be more susceptible to COVID-19, and had a higher rate of ICU admission than children over 1 year of age. Other epidemiological differences between pediatric patients and adult patients include a higher rate of familial infection, a longer incubation period (6.5 days vs. 5.4 days in adult) and a higher rate of detection of the virus in feces [82].

### Specific issues relevant to pediatric surgery

On April 23, 2020, the Japanese Society of Pediatric Surgeons posted guidelines and COVID-19 news in relation to pediatric surgery. Striking data published on April 5 from Wuhan stated that the COVID-19-related mortality rate of adult patients undergoing major surgeries was as high as 20.5%, subsequent to the postoperative development of ARDS. Although the majority of children with COVID-19 are asymptomatic, as the virulence is still largely unknown, it would be wise to perform a preoperative PCR test, not only to protect healthcare professionals but also to safeguard the patient from postoperative COVID-19-related mortality [83].

As the community-acquired infections rate increases, we will encounter more COVID-19-positive patients with common disease entities such as appendicitis. It would be prudent to simulate the response and prepare for the management of such patients with acute conditions in advance. Whether an immediate surgical approach or non-surgical management should be implemented for patients with uncomplicated appendicitis depends on the accessibility of COVID testing, PPE supplies, and the regional prevalence of COVID-19, and thus remains a matter of debate [84].

As of April 30, 2020, there were few published articles clearly demonstrating that children with underlying disease are more susceptible to COVID-19, despite the published evidence of its severity in adult patients with underlying diseases, including oncologic disease [85], cardiac disease [86] and organ transplantation [87]. A judicious approach should be taken to avoid mistakes in the standard differential diagnosis when treating patients with underlying disease, without being too fearful of COVID-19.

Although vertical transmission seems unlikely [88], once neonates are infected, COVID-19 RNA can be detected in all of their clinical specimens, including blood, urine, stool and saliva along with upper airway tract specimens, suggesting that extraordinary caution must be taken to prevent transmission to healthcare professionals if these neonates require surgical intervention. The longer time to detoxification in feces than in throat swabs in children with COVID-19 (2–4 weeks vs. 1 week, respectively) [89] should raise awareness among pediatric surgeons, especially those dealing with anorectal malformation, Hirschsprung’s disease and other lower gastrointestinal diseases.

### Impact of the COVID-19 pandemic on pediatric surgery in Tokyo

As of May 3, 63 cases of children aged < 10 years and 70 cases of children aged < 10–20 years had been identified in the Tokyo metropolitan area, accounting for 2.97% of the total 4477 PCR-confirmed COVID-19 cases. In the Tokyo metropolitan area, there are two designated pediatric hospitals, the National Center of Child Health and Development and Tokyo Metropolitan Children’s Medical Center, as well as 11 university hospitals with certified surgical programs accommodating a pediatric population of 1.5 million. Keio University Hospital, a university hospital that initiated major changes for COVD-19, implemented an incident command structure [90] in mid-March 2020, which facilitates rapid communication between hospital leadership and frontline professionals. We also shared our experience and information with colleagues in two pediatric hospitals to develop a collaborative process that adapts to rapid and ongoing change. Table 2 shows the timeline of changes in clinical practice policy for pediatric surgery at three major hospitals. The basic rules for surgical triage proposed by the American College of Surgeons were extrapolated with certain modifications. As of April 2020, there were some differences in screening policies among the centers, but we have still to identify which strategy represents the best practice.

**Table 2:** Timeline of clinical practice policy for pediatric surgery

	Keio Children’s Hospital and Perinatal Center.	National Center for Child Health and Development	Tokyo Metropolitan Children’s Medical Center
Surgical Triage Policy (Department of Pediatric Surgery)	All elective cases cancelled since Apr 1	Reduction of elective cases since Mar 31, all elective cases cancelled since Apr 6	Reduction of elective cases 30% since Apr 2, all elective cases cancelled since Apr 17
Actual number of surgical cases performed between weeks of Apr 6–Apr 25	3 urgent cases	25 cases (8 emergency and urgent cases only)	33 cases (4 emergency and mostly urgent cases)
Preoperative Surveillance	PCR for All + Chest CT if > 7 of age since Apr 6	PCR only for suspect cases as of Apr 30	PCR only for suspect cases as of Apr 30
Surveillance of PCR for admission	^a^All since Apr 8	Only suspect cases as of Apr 30	Only suspect cases as of Apr 30
Reconfiguration of ward	3 isolated rooms for COVID-19	One designated floor for suspect and confirmed COVID-19	28 isolated beds +2 PICU for COVID-19
Rearrangement of surgical team	Since Apr 5	Since Apr 13	Since Apr 6
Ambulatory care	Triage Since Mar 28 and Telemedicine since Apr 1	Triage and Telemedicine since Apr 13	Triage and Telemedicine since Mar 6

^a^All tested subjects stay home until the results are available. If urgent, the subject is isolated in one of three rooms for a single suspect case until the result is available

4. Summary

As COVID-19 affects children differently from adults, pediatric surgeons must be fully aware of the child-specific issues relevant to COVID-19, as outlined above. Because we are facing a nationwide pandemic crisis, the establishment of a rapid communications system between hospital leadership and frontline professionals is of paramount importance for optimizing the medical function under limited medical resources.

## Transplantation

As COVID-19 patients have been encountered in organ transplantation hospitals, hospital infections are a major concern, not only because organ transplant recipients, who are immunosuppressed are highly vulnerable to COVID-19 complications, but also the transmission of COVID-19 from donors must be prevented. Thus, we must take extreme care in the transplantation process and in post-transplantation management of patients. Preventing the infection of medical staff and those involved in deceased donor transportation to the donation facility is also of paramount importance.

Before a state of national emergency was declared, The Japan Society for Transplantation (JST) formed a task force committee against COVID-19 and published the first for general awareness and restriction of transplantation, on March 6, 2020 [91]. Live-saving transplantation such as heart, lung, and liver transplants could be continued with informed consent about the risks of COVID-19. However, it was recommended that kidney, pancreas, and intestinal transplantation be suspended. SARS-Cov2 nucleic acid testing (NAT) is performed, and COVID-19 or exposure to COVID-19-related risk factors is checked in every deceased donor. Whenever possible, organ recovery should be performed by the local procurement team and the transplant team should minimize exposure to any COVID-19-related risk. All living donors and candidates for kidney, lung and liver transplants are directed to stay home or in hospital for 14 days prior to the transplantation. In hospitals where NAT is available, it is recommended on Day-14 and Day-1 for both donors and recipients. Chest CT screening is also recommended for both donors and recipients before transplantation. The JST recommends reducing the frequency of outpatient visits and supplying additional medications to recipients in case of a lockdown in their area of residency. The JST also recommends telephone consults and delivery of medications to patients in their homes to reduce the risk of exposure to COVID-19.

At the beginning of April, the JST started the patient registry on COVID-10 recipients, not only for epidemiologic data but also for providing treatment options to save them. The JST published the second guidelines with Q&A for safe deceased organ donation, on April 11, 2020, and also provided a risk evaluation table of COVID-19 for the donor under brain death and after cardiac death [92].

The JST published the third guidelines with clinical questions on the treatment of transplantation recipient with COVID-19 on May 6, 2020 [93, 94]. These were based on expert opinions and publications current at the time. The treatments recommended in the guidelines and clinical questions should be restricted not only to the transplantation and infection specialists, but also in the hospitals where intensive care facilities can be on stand-by for rapid deterioration of COVID-19 recipients.

On May 16, 2020, there were eight transplant recipients with COVID-19 in Japan (seven kidney transplant recipients and one heart transplant recipient). Not all their prognoses are registered yet, but at the time, there had been a report of one death and three recipients treated early with favipiravir resulting in rapid improvement of their symptoms. The limited experience of these cases supports early treatment by effective anti-viral drugs before the cytokine storm [95, 96].

The homepage of the JST website cites COVID-19-related new information for transplantation professionals, which is updated daily [97].

## Abbreviations

AGPs: aerosol-generating procedures, extracorporeal membrane oxygenation
ECMO: Novel coronavirus disease 2019
COVID-19: Personal protective equipment, PPE
